# Bismuth nanoparticles obtained by a facile synthesis method exhibit antimicrobial activity against *Staphylococcus aureus* and *Candida albicans*

**DOI:** 10.1186/s42490-020-00044-2

**Published:** 2020-10-14

**Authors:** Roberto Vazquez-Munoz, M. Josefina Arellano-Jimenez, Jose L. Lopez-Ribot

**Affiliations:** 1 The University of Texas at San Antonio, San Antonio, TX 78249 USA; 2 The University of Texas at Dallas, Richardson, TX 75080 USA

**Keywords:** Nanoantibiotics, Bismuth nanoparticles, Biofilms, Antimicrobial nanomaterials, Bacteria, Fungi

## Abstract

**Background:**

Bismuth compounds are known for their activity against multiple microorganisms; yet, the antibiotic properties of bismuth nanoparticles (BiNPs) remain poorly explored. The objective of this work is to further the research of BiNPs for nanomedicine-related applications. Stable Polyvinylpyrrolidone (PVP)-coated BiNPs were produced by a chemical reduction process, in less than 30 min.

**Results:**

We produced stable, small, spheroid PVP-coated BiNPs with a crystalline organization. The PVP-BiNPs showed potent antibacterial activity against the pathogenic bacterium *Staphylococcus aureus* and antifungal activity against the opportunistic pathogenic yeast *Candida albicans*, both under planktonic and biofilm growing conditions.

**Conclusions:**

Our results indicate that BiNPs represent promising antimicrobial nanomaterials, and this facile synthetic method may allow for further investigation of their activity against a variety of pathogenic microorganisms.

## Background

Bismuth (symbol: Bi, Z = 83, A = 208.98) is a metallic element, non-toxic for humans (Lethal Intake > 5–20 g/day/Kg, for years) [1, 2]. Bismuth is insoluble in water but soluble in some organic solutions. The water-solubility and lipophilicity of bismuth can be enhanced when it is complexed with lipophilic molecules [3] and its biocompatibility is increased when it is chelated with hydroxyl or sulfhydryl groups. Bismuth compounds have been used in medicine for more than two centuries [4]. Bismuth subsalicylate has been utilized to treat diarrhea-related ailments since the 1900s [5], and most recently, bismuth compounds have been used in computed tomography imaging and anti-cancer therapy [6, 7]. In the U.S., around 30% of the bismuth compounds are used for cosmetic and pharmaceutical applications [4].

Several bismuth compounds display antibacterial and antifungal activity [8, 9] and can outperform the inhibitory activity of conventional antibiotics [10]. When bismuth is thiolated, such as in the case of bismuth-thiols (BTs) complexes, its antimicrobial activity is further enhanced [11]. Bismuth-dimercaptopropanol (bismuth-BAL) and other BTs display anti-biofilm activity [12]. Additionally, the thiolation of bismuth complexes increases their stability [13]; however, BTs stability is still relatively low when compared to other antimicrobial agents [14].

The stability and antimicrobial activity of bismuth can be improved using nanotechnological approaches, as the detailed control of size, structure, and environment define the structural and physicochemical traits of nanostructures. Phan et al suggested that nanomaterials may display greater stability than their precursors under some specific conditions [15]. Also, the capping agents provide stability to nanoparticles [16]. Stability is critical for controlling the BTs potential toxicity [17] and for extending their shelf-life. In the last two decades, metallic elements with known –or potential- antimicrobial properties (silver, copper, and titanium, among others) have been used for synthesizing antimicrobial nanomaterials (nanoantibiotics) [18, 19].

**Fig. 1:**
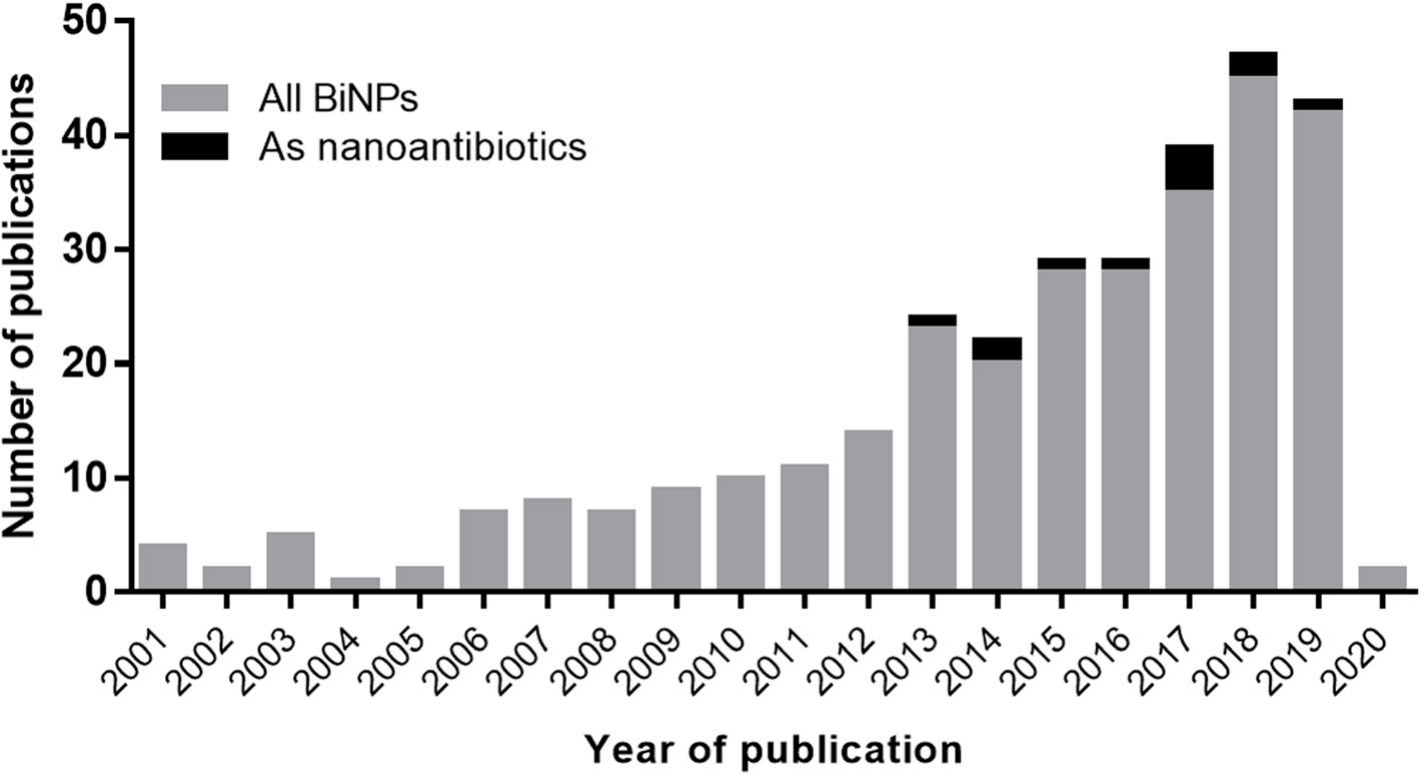
Research of BiNPs in recent literature. BiNPs as antimicrobial agents remain barely researched. According to Web of Science, approximately 300 studies on BiNPs have been published in the last 20 years, from which only 12 are related to their antimicrobial activity. “The search parameter in the Web of Science (WoS) search was: “Bismuth nanoparticles” in title, abstract, and keywords. Then, the results were filtered by “antimicrobial” OR “antibacterial” OR and “antifungal””

Although some nanoantibiotics have been successfully transitioned to the market [20, 21], some promising metallic elements, such as bismuth, remain under-researched, even though some early reports indicate that bismuth nanoparticles (BiNPs) display promising antimicrobial activity on bacteria, fungi, and protozoan [3, 22]. A recent review from Shahbazi et al. [23], describes the diverse biomedical applications of bismuth compounds, including the antimicrobial properties of bismuth-based nanoparticles. According to Web of Science, 300 studies on BiNPs have been published in the last 20 years, yet only 12 are related to their antimicrobial activity (Fig. 1). This scarceness on research may be due to the difficulties in synthesizing BiNPs and their low stability under culture conditions.

Most of the current protocols for synthesizing biologically suitable BiNPs cannot be replicated in non-specialized laboratories. Some methods require specialized equipment, such as gamma irradiators [24], laser ablation [25]; or controlled conditions, such as inert atmosphere [26], vacuum [27], or high temperatures [28, 29]. Also, some protocols require a long-time synthesis [24], or several intermediate steps [30]. Although biosynthesis -green chemistry- has been explored, it is not easily replicable in non-specialized laboratories [31, 32]. In general, BiNPs obtained using the above-mentioned protocols display an aspect ratio close to 1; although polyhedral, rod-like, and triangular shapes have also been described, and their sizes range from 1.7 nm to 1000 nm (1 μm). Most recently our group has described a method for the fast, facile, and inexpensive synthesis of BiNPs using a simple chemical reduction process, which results in the generation of small nanoparticles with an aspect ratio close to 1. This novel methodology does not require the use of sophisticated equipment, is easy to replicate, and can be readily implemented in non-specialized laboratories [33]. The main objective of this work was to elucidate the antimicrobial activity of these easy-to-synthesize BiNPs against *Staphylococcus aureus*, a pathogenic bacterium, and *Candida albicans*, an opportunistic, dimorphic yeast; and in the process increasing the research on this new type of nanoantibiotics.

## Methods

### Reagents and nanomaterials

2,3-Bis(2-methoxy-4-nitro-5-sulfophenyl)-2H-tetrazolium-5-carboxanilide salt (XTT), menadione, and phosphate Saline Buffer (PBS) from Sigma-Aldrich (MO). Osmium Tetroxide (OsO_4_) and Glutaraldehyde from Ted Pella; and PrestoBlue™ Cell Viability Reagent from InVitrogen. 1% OsO_4_ and 2.5% glutaraldehyde solutions were prepared in Milli Q water. A 10% PrestoBlue solution was prepared in BPS.

#### Nanoantibiotics

PVP-coated bismuth nanoparticles were produced by an easy-to-synthesize chemical reduction protocol recently reported by our group [33]. Briefly, in a pre-warmed glycine solution, bismuth nitrate salts were added, and the pH was raised to 9. Then, BAL and PVP solutions were consecutively added to the bismuth solution. Finally, a NaBH_4_ solution was added, dropwise, in two batches three minutes apart from each other. The suspension was kept on vigorous stirring for 10 min.

### Characterization of the PVP-BiNPs

In a previous report, we demonstrated the presence and essential characteristics of the BiNPs, via Transmission Electron Microscopy and Energy Dispersive X-ray Spectroscopy, as well as the ƺ-potential via Dynamic Light Scattering Spectroscopy [33]. In this study, we expanded the characterization of the BiNPs to include different rounds of synthesis, as follows: *High-Resolution Transmission Electron Microscopy* (HR-TEM) for determining the single-particle structural lattice, using Selected-Area Electron Diffraction (SAED) analysis. The PVP-BiNPs, deposited on Type-B carbon-coated copper grids (Ted Pella Inc.), were examined in a JEOL 2010-F HR-TEM (Jeol Ltd.), with 200 kV accelerating voltage. *Dynamic Light Scattering analysis* to determine the hydrodynamic size, using a Zetasizer Nano ZS (Malvern Panalytical). PVP-BiNPs were diluted in Milli Q water and then transferred to a DTS1070 cell for the analysis. *UV-Vis spectroscopy* to follow the transition from bismuth (III) ions to the PVP-BiNPs, via the UV-Visible absorbance profile, in 10 nm steps, collected in a Genesys10 UV-Vis spectrophotometer (Thermo Fisher), from 200 to 600 nm.

### Antimicrobial susceptibility assays

#### Strains and culture conditions

In this study, the Gram-positive bacterium USA200 methicillin-sensitive *S. aureus* (MSSA) UAMS-1 strain and the dimorphic yeast *C. albicans* strain SC5314 were used. For growth, *S. aureus,* inoculated in tryptic soy broth (TSB) (BD Difco, MD) and *C. albicans*, inoculated in Yeast extract Peptone Dextrose (YPD) broth, were incubated overnight in an orbital shaker at 35 °C. The antimicrobial activity of BiNPs was assessed in the planktonic and biofilm formation stages, as follows:

#### BiNPs antimicrobial activity on the microbial planktonic cells

We followed the CLSI guidelines to evaluate the susceptibility to the BiNPs, with slight modifications. For *S. aureus* the CLSI M07 [34] guidelines were followed; whereas for *C. albicans* the CLSI M27 [35] guidelines were used. Briefly, overnight microbial cultures were washed twice in PBS; adjusted for a final concentration of 10^6^ cells mL^− 1^ in MH broth for *S. aureus*; and to 10^3^ cells mL^− 1^, in RPMI culture media, for *C. albicans*. Then, 50 μL of each microbial strain were transferred to 96-multiwell plates. Subsequently, bismuth compounds [BiNPs, bismuth-BAL and Bi(NO_3_)_3_] were prepared in a two-fold dilution series, for a final concentration range from 0.5 to 256 μg mL^− 1^ (from 2.39 μM to 1.22 mM), and 50 μL were added to the multiwell plates with the microbial cells. Later, the multiwell plates were cultured at 180 rpm; at 37 °C for 24 h. The Minimal Inhibitory Concentration (MIC) was determined as the concentration at which no turbidity was detected.

#### Assessment of the BiNPs antibiofilm activity on *S. aureus*

The ability of the BiNPs to inhibit biofilm formation by *S. aureus* was evaluated following a protocol previously published by our group with minor modifications [36]. Briefly, a “*biofilm broth*” was prepared as follows: 45% Tryptic-Soy Broth (BD Difco, MD), 45% Brain-Hearth Infusion broth (BD Difco, MD), and 10% Bovine Fetal Serum (BD Difco, MD). Bacterial cells were washed twice in PBS and adjusted to 10^8^ cells mL^− 1^ in the *biofilm broth* and transferred to the 96-multiwell plates. Bismuth compounds were prepared in a two-fold dilution series, in the *biofilm broth*, for a final concentration range from 0.5 to 256 μg mL^− 1^ (from 2.39 μM to 1.22 mM) and transferred to the multiwell plates with *S. aureus*. The plates were cultured at 37 °C for 24 h. After the incubation time, the biofilms were washed with PBS and the Presto Blue™ was added, and the multi-well plates were read spectrophotometrically as previously described by our group [37].

#### Assessment of the BiNPs antibiofilm activity on *C. albicans*

The anticandidal activity of BiNPs, for preventing the biofilm formation, was evaluated using the methods previously reported by our group [38], with minor modifications. *C. albicans* cells from overnight cultures were washed in PBS, adjusted to × 10^6^ cells mL^− 1^ in RPMI culture media, and transferred to the 96-multiwell plates. Then, BiNPs two-fold dilution series were prepared in RPMI, for a final concentration range from 0.5 to 256 μg mL^− 1^, and transferred to the plates with *C. albicans*, and then cultured at 35 °C for 24 h. Post-incubation, *C. albicans* biofilms were washed with PBS, then XTT/menadione was added. The absorbance of XTT was collected in a Benchmark Microplate Reader (Bio-Rad Inc), at λ = 490 nm.

To generate the dose-response curves, the normalized results from the absorbance readings (from *S. aureus* and *C. albicans*) were fit to the variable slope Hill equation (for the nonlinear drug dose-response relationship) using Prism 8 (GraphPad Software Inc). Then, the BiNPs IC_50_ was calculated. The IC_50_ was established as the concentration of BiNPs that reduce the 50% of the bacterial growth. To ensure the reproducibility of the antimicrobial activity from the different rounds of BiNPs syntheses and the bismuth compounds, we used two biological replicates (multi-well plates) with two technical replicates within each plate.

### Ultrastructural analysis

To assess the impact of the BiNPs on the biofilm structure and cell morphology of *S. aureus* and *C. albicans*, the samples were analyzed via Scanning Electron Microscopy (SEM). Briefly, BiNPs-treated and control (untreated) samples were washed with PBS, fixed with a 2% glutaraldehyde solution for 60 min, and then stained and post-fixed with a 1% Osmium tetroxide solution, for 30 min at 4 °C. Next, samples were washed with PBS and dehydrated using an ascending concentration ethanol series, up to 100%, and left to dry overnight. Finally, the samples were coated with gold in a Sputter Coater SC7620 (Quorum Technologies), for 25 mA for 3 min. The gold-coated samples were observed in a TM4000Plus Scanning Electron Microscope (Hitachi Inc.), with a magnification 500 and 2500x, using a 10 KeV voltage.

## Results and discussion

### Characterization of the BAL-mediated PVP-BiNPs

#### High-resolution transmission electron microscopy (HR-TEM)

In a previous report, HR-TEM revealed the morphology of the BiNPs [33]. For this work, an extended characterization via HR-TEM was performed in other rounds of syntheses. Our analyses revealed that the shape of the nanoparticles displayed an aspect ratio close to 1, although few specific shapes (i.e. polyhedrons) were observed (supplementary materials, Fig. s1). The average diameter of the nanoparticles is 8.4 nm ±6.7 nm, ranging from 1.7 nm to 44.4 nm (*n* = 1159) (supplementary materials, Fig. s1, inset). Approximately, 86.1% of the particle size was below 15 nm. Some larger individual nanoparticles (50–100 nm) and clusters of nanoparticles were also observed. The cause of the formation of large individual nanoparticles and clusters may be due to the formation of “fast nucleation hotspots” where the NaBH_4_ is added into the solution.

**Fig. 2:**
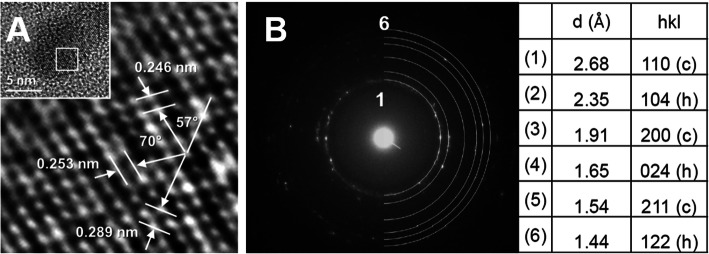
Characterization of the BAL-mediated PVP-BiNPs. **a** Our HR-TEM analysis from a single particle confirms their crystalline organization, whereas the (**b**) Electron Diffraction Pattern reveals their crystalline lattice as a cubic and hexagonal organization

The single-particle HR-TEM analysis revealed the crystallinity of the PVP-BiNPs. Lattice distance measurements agreed with reported low-index planes for crystalline Bi with cubic, hexagonal, and orthorhombic cell. The crystalline lattice depicted in Fig. 2a shows distances of 0.289, 0.253, and 0.246 nm, which suggest an orthorhombic cell (0.290 nm is the distance reported for the (2 1–1) plane in the orthorhombic cell) or misorientation. The SAED from the BAL-mediated PVP-BiNPs showed the appearance of a clear ring diffraction pattern, which confirmed the formation of crystalline bismuth nanostructures. Interestingly, the experimental d-spacing corroborates that BiNPs display a mixed arrangement, conformed by cubic and hexagonal phases. The d-spacing for the cubic lattice were 2.68, 1.91, and 1.54 Å (hkl 110, 200, and 211, respectively); whereas for the hexagonal lattice were 2.35, 1.65, and 1.44 Å (hkl 104, 024, and 122, respectively) (Fig. 2b), according to the data from the standard powder diffraction cards of the Joint Committee on Powder Diffraction Standards (JCPDS), bismuth files numbers #26–0214 (cubic phase), and #44–1246 (hexagonal phase).

#### Dynamic light scattering spectroscopy

The PVP-BiNPs hydrodynamic size is 22.5 ± 0.06 nm. The hydrodynamic size is greater than the metal core size observed on TEM images (22.5 nm vs 8.6 nm respectively). The increase in the hydrodynamic size may correspond to the 10 K PVP coating chain-like molecules, whose structures extend when hydrated under the aqueous environment. The PVP coating improves BiNPs stability. HR-TEM images reveal that BiNPs are individual structures; however, they may be clustering over time due to the interactions of the organic compounds in the suspension.

#### UV-Vis characterization

**Fig. 3:**
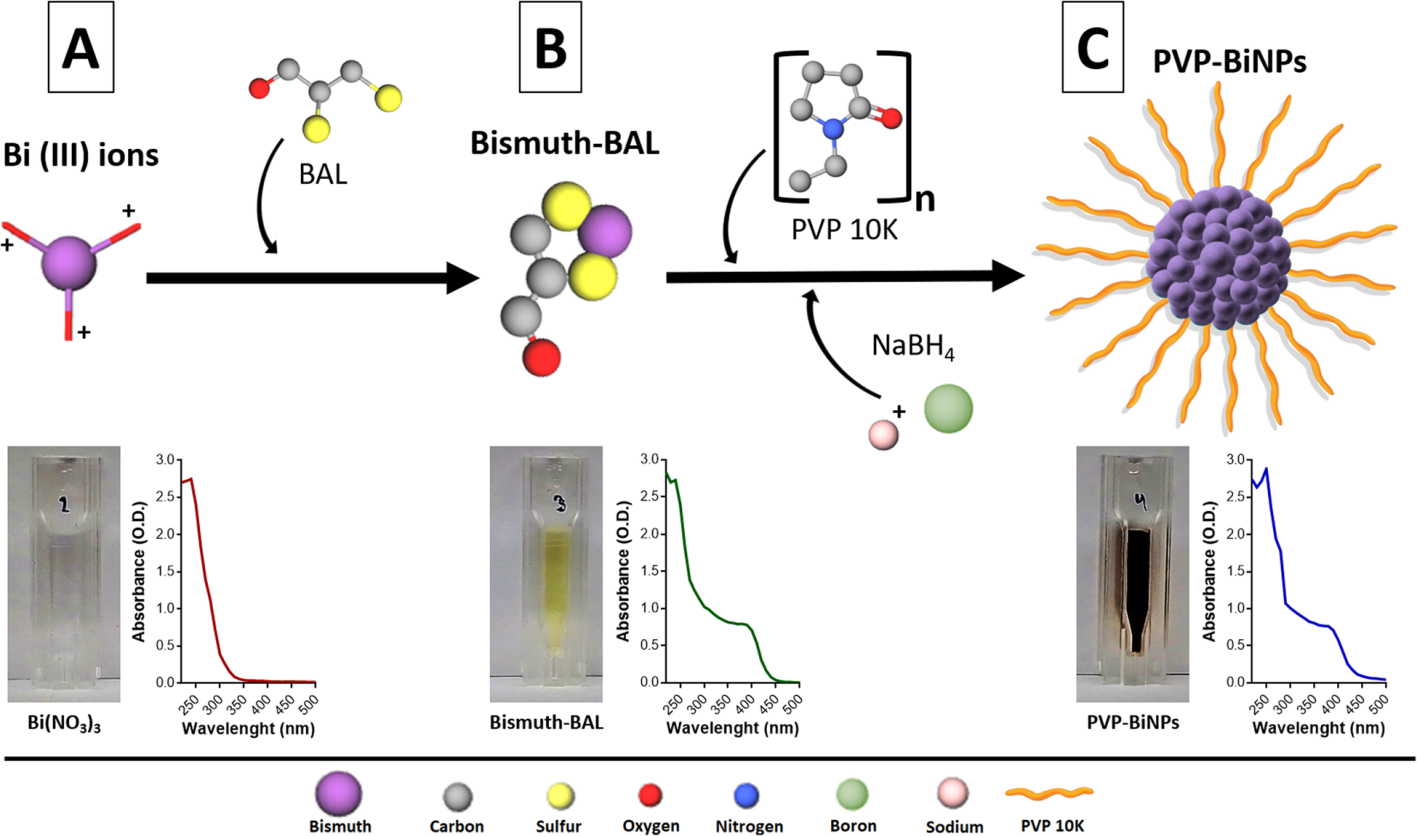
Proposed mechanism of synthesis for the formation of BiNPs. Bismuth (III) ions, solubilized in a glycine solution (**a**), interact with BAL, leading to the formation of the bismuth-BAL complex (**b**). Finally, NaBH_4_ induces the generation of PVP-BiNPs (**c**). Hydrogens atoms were not included in the molecular models for clarity. Molecules were built using the GLmol engine at http://molview.org/

The bismuth compounds displayed distinct absorbance profiles. The bismuth (III) ions profile (diluted in glycine) displayed an absorbance profile with a maximum at λ = 240 nm, whereas the bismuth-BAL complex displayed a decreasing profile with a flat absorbance profile, from λ = 340 to 390 nm, with a rapid decline λ = 400 nm. In contrast, the BiNPs absorbance profile showed a surface plasmon with a shoulder at λ = 250 nm, close to the reported by other authors [39], and a shoulder at λ = 270 nm. Also, a flat profile was observed at λ = 340 with a decrease at λ = 380 nm. The difference observed in the absorbance profiles demonstrates the chemical transition from the bismuth (III) ions to the bismuth-BAL complex and then to the bismuth nanoparticles (Fig. 3**, bottom**). As seen in nanoparticles from other elements, the absorbance profile is determined by the surface physicochemical traits of the nanoparticles, such as shape and size distribution and the capping agents, among others [40]. In contrast to the AgNPs, a “typical” UV-Vis absorbance profile for BiNPs has still not been suggested. According to the literature, different bismuth nanoparticles exhibit spectra profiles with maximum peaks at bands in different locations, such as λ = 275 nm and λ = 360 nm [41], λ = 281 nm [42], 253 nm [39], and λ = 313 nm [43]. Ma et al reported that BiNPs with a size below 10 nm did not display a UV-Vis absorbance profile. However, our small BiNPs did show a profile (average size < 10 nm), yet, the absorbance profile may be from the BiNPs larger than 10 nm.

### Mechanism of BAL-mediated PVP-BiNPs synthesis

We used the bottom-up methodology approach for producing the BAL-mediated synthesis of the PVP-BiNPs. In a previous work, we described the procedure for synthesizing the nanoparticles [33]. Here, we propose the potential interactions between the precursors, aiming to describe the mechanism that leads to the formation of the PVP-BiNPs. The water-insoluble bismuth nitrate readily dissolves in the glycine solution, allowing the release of bismuth (III) ions (Fig. 3**, segment A**), but the induction of an alkaline environment reduces the solubility of bismuth (III) ions. Yet, the alkaline environment allows dimercaptopropanol (BAL) to rapidly sequester the bismuth (III) ions, favoring the formation of the highly soluble bismuth-BAL complex (the solution changes from white to yellow, and the absorbance profile changes) (Fig. 3**segment B**). PVP K-10 -a dispersant, mild reducing agent-, slowly initiates the formation of the nanoparticles, while regulates their size and shape. Moreover, PVP interacts chemically with bismuth [42], leading to the formation of a PVP coat on the nanoparticles. Although different biomolecules have been used as stabilizers for synthesizing BiNPs, such as starch [44]; PVP-K10 was selected due to the multiple properties that confer to the nanoparticles –negative charge, stability, and low toxicity- [45–47]. NaBH_4_ -a strong reducing agent- speed up the synthesis process. The rapid formation of the BiNPs is revealed by the color change of the reacting mixture (from yellow to black), and by a final transition on the UV-Vis absorbance (Fig. 3**segment C**). The change of color to black is attributed to the increasing presence of –nanostructured- elemental bismuth, due to the shift of the oxidation state during the reduction of bismuth (III) ions [41]. The HR-TEM single-particle analysis and the DLS spectroscopy confirm the formation of bismuth nanoparticles, tentatively coated by PVP, as the hydrodynamic diameter is larger than the metallic BiNPs diameter.

### Stability of the PVP-coated BiNPs

The stability of bismuth nanoparticles in aqueous suspensions depends on several factors, such as pH [48], exposure to air [49], capping agent [50], and the bismuth-thiol bonding [11, 13]. Thiolation increases the bismuth solubility and stability [13]; yet, the stability of bismuth-thiol complexes is still relatively low, as they remain in suspension for short periods [14]. Our synthesis method [33] enhances the BiNPs stability when compared with the bismuth (III) ions and the bismuth-BAL complex, under the same conditions of temperature, exposure to atmospheric air, and changes on pH. Preparations of 15 mM bismuth nitrate and 15 mM bismuth-BAL complex precipitated within a few days; in contrast, the BAL-mediated PVP-coated BiNPs are stable for at least 11 weeks (Fig.s2, supplementary materials). This increase in stability is an improvement over other processes for producing PVP-BiNPs, as it has been reported that PVP-coated BiNPs precipitate in less than 4 days [50]; Also, some studies show that BiNPs in aqueous suspensions are rapidly dissolved if the pH is out of the 8–10 range [48] or when exposed to air [51], yet our BiNPs remained stable for hours when dissolved in MilliQ water (pH 7) and when exposed to air. In our protocol, both solubility and stability of bismuth are increased due to the addition of BAL, leading to more stable BiNPs. The BAL-mediated synthesis of PVP-BiNPs kept the stability of nanoparticles even though the synthesis was performed under atmospheric air conditions.

Moreover, we tested variations on the synthesis method and found that PVP-BiNPs without BAL were unstable, precipitating in less than a week, whereas BiNPs without glycine or PVP were highly unstable, precipitating within minutes after the synthesis. These findings suggest that BiNPs stability is related to the bismuth (III) ions availability for the synthesis process (increased solubility) and capping molecules, with PVP acting as a capping/dispersant agent [47].

### BiNPs display antibacterial and antifungal activity on the planktonic and biofilms stages

**Fig. 4:**
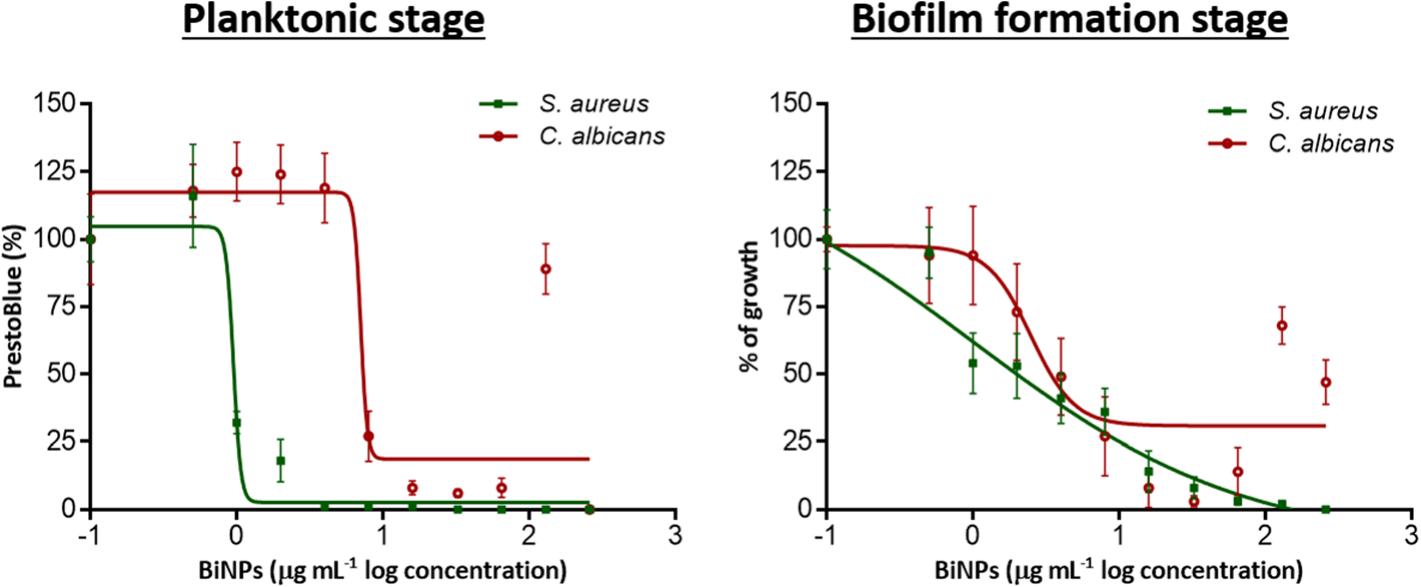
Antimicrobial activity of the BiNPs. Dose-response curves of the BiNPs antimicrobial activity against the bacterium *S. aureus* and the yeast *C. albicans*. Planktonic cells (green line) and Biofilms formation stage (red lines)

PVP-BiNPs display strong antimicrobial activity against the planktonic cells *S. aureus* and *C. albicans* (Fig. 4a). The BiNPs MIC against the planktonic cells of *S. aureus* was 1 μg mL^− 1^. The antibacterial activity of these BiNPs is similar or better than the BiNPs activity reported before in the literature. Previously reported BiNPs MIC values ranged from 1.05 μg mL^− 1^ for *S. mutans* and *S. gordonii* to 1500 μg mL^− 1^ for the MRSA strain [3, 52]. Also, the antimicrobial activity of the resulting BiNPs is similar or better than several antimicrobial drugs, such as Vancomycin or Oxacillin (MIC = 1.5 μg mL^− 1^) and Ceftaroline (MIC = 0.5 μg mL^− 1^) [53]. Likewise, the BiNPs antimicrobial activity outperforms the antibacterial activity of silver nanoparticles (AgNPs) (MIC = 4 μg mL^− 1^) [54] and other metallic nanoparticles, such as copper (MIC≥28.6 μg mL^− 1^) or zinc (MIC≥85.8 μg mL^− 1^) [55].

For *C. albicans*, the BiNPs MIC was 16 μg mL^− 1^. As far as the authors know, the antifungal activity of BiNPs has only been addressed in one other study, also against *C. albicans*, with a MIC equivalent to 2.09 μg mL^− 1^ [3]. When compared with antifungals, the BiNPs antimicrobial activity for *C. albicans* is parallel to azole antifungal agents but less potent than amphotericin B or echinocandins [56, 57]. In contrast to their antibacterial activity, the BiNPs antifungal activity seems to be lower when compared with AgNPs (MIC = 2 μg mL^− 1^) [54]; however, they are more potent than other nanoantibiotics, such as copper nanoparticles (MIC≥150 μg mL^− 1^) [58].

The synthesized PVP-BiNPs were also capable of inhibiting biofilm formation by both *S. aureus* and *C. albicans* (Fig. 4b). In the case of *S. aureus* biofilms, the IC_50_ was 1.06 μg mL^− 1^, which is among the most potent activity of BiNPs reported against the biofilm formation phase on bacteria. Baddidery et al reported that 2.61 μg mL^− 1^ prevents the cell attachment on a polycarbonate membrane [59]. In contrast, Dalvand showed that even concentrations of BiNPs as high as 1500 μg mL^− 1^, inhibited the MRSA biofilms only by 16% [52]; Moreover, Hernandez-Delgadillo reported that a BiNPs concentration of 10,000 μg mL^− 1^ was required to prevent the biofilm formation on *Enterococcus faecalis* [60]. BiNPs display better antibiofilm activity than silver nanoparticles (IC_90_ = 15 μg mL^− 1^) [61]. When compared with antibacterial drugs, the BiNPs exhibit comparable antibiofilm activity to that of vancomycin (IC_100_ = 2 μg mL^− 1^) and linezolid (IC_100_ = 4 μg mL^− 1^) [62].

Regarding *C. albicans*, the calculated IC_50_ value for BiNPs against biofilms formed by this fungus was 7.9 μg mL^− 1^, which interestingly is very close to its value under planktonic conditions As far as the authors know, the antibiofilm activity of BiNPs only has been addressed in one other study, where a concentration of 418 μg mL^− 1^ was required to inhibit the *C. albicans* biofilm [63].. When compared with BiNPs, AgNPs display better antibiofilm activity (IC_50_ = 0.089 μg mL^− 1^) [64]. The anticandidal activity of BiNPs, compared to antifungal agents, is better than Fluconazole (IC_50_ > 1024 μg mL^− 1^), but lower than Amphotericin B (IC_50_ > 1 μg mL^− 1^) and the echinocandins [65, 66]. In Table S1 (supplementary materials) the antimicrobial activity of our BiNPs is compared with other BiNPs reported in the literature, against the planktonic and biofilm stages in bacteria and fungi.

Moreover, the antibacterial effect of Bi(NO_3_)_3_ and the bismuth-BAL complex was assessed against the planktonic stage and during the biofilm formation phase of *S. aureus* and *C. albicans*. For *S. aureus*, the antibacterial activity of the bismuth-BAL complex equals that of the BiNPs. In contrast, bismuth (III) ions have the lowest performance. For *C. albicans*, BiNPs displayed the highest antifungal activity, followed by the bismuth (III) ions, whereas the bismuth-BAL complex exhibited the lowest activity. The activity of all bismuth compounds under planktonic and biofilm growing conditions is summarized in Table 1, whereas the corresponding dose-response curves are in the supplementary materials section (Fig. s3). As suggested by Domenico et al [67], the bismuth-BAL complex displays better antimicrobial activity than the Bi(NO_3_)_3_ salts. The antibacterial activity of different bismuth compounds against the bacterial planktonic stage and biofilm has been reported by different groups [8, 12].

**Table 1:** Antimicrobial activity of bismuth compounds (μg mL^− 1^) on the planktonic cells and biofilm stages of *S. aureus*

Phase/strain	Treatments Inhibitory concentration		
	BiNPs	Bismuth-BAL complex	Bismuth (III) ions
Planktonic cells^a^			
*S. aureus*	0.9	1.1	161.2
*C. albicans*	7	57.4	8
Biofilm formation^b^			
*S. aureus*	1	2.1	31
*C. albicans*	2.5	> 256	8.5

^a^ Minimal Inhibitory concentration (MIC)

^b^ Calculated IC_50_

Furthermore, since the antimicrobial activity of BiNPs has been scarcely addressed, the mechanism of action has not been elucidated. Both BiNPs precursors –bismuth (III) ions and bismuth-BAL complex- display antimicrobial activity against both strains, therefore it is likely that the fundamental mechanisms of action of BiNPs are related to their precursors. The mechanisms of action of bismuth (III) ions are partially known. Ionic bismuth binds to glutathione, which improves its translocation inside of cells, from where it targets proteins and inhibit enzymes, such as urease and fumarase [68, 69]. Regarding the bismuth-BAL complex, the mechanisms related to their antimicrobial activity are not fully understood. However, it is known that bismuth-thiols increase the permeability of the bacterial cell membrane [70]. Also, in the case of *S. epidermalis*, bismuth-thiol complexes reduce the glycocalyx production on the capsules [67]. It is highly likely that BiNPs release bismuth (III) ions with antimicrobial activity; though, due to the evident difference in the antimicrobial activity between BiNPs and bismuth (III) ions, the nanostructured arrangement of the BiNPs must be playing an unknown key role for enhancing the activity. So far, the mode of action of the BiNPs remains to be uncovered.

### The BAL-mediated PVP-BiNPs alter the biofilm ultrastructure

**Fig. 5:**
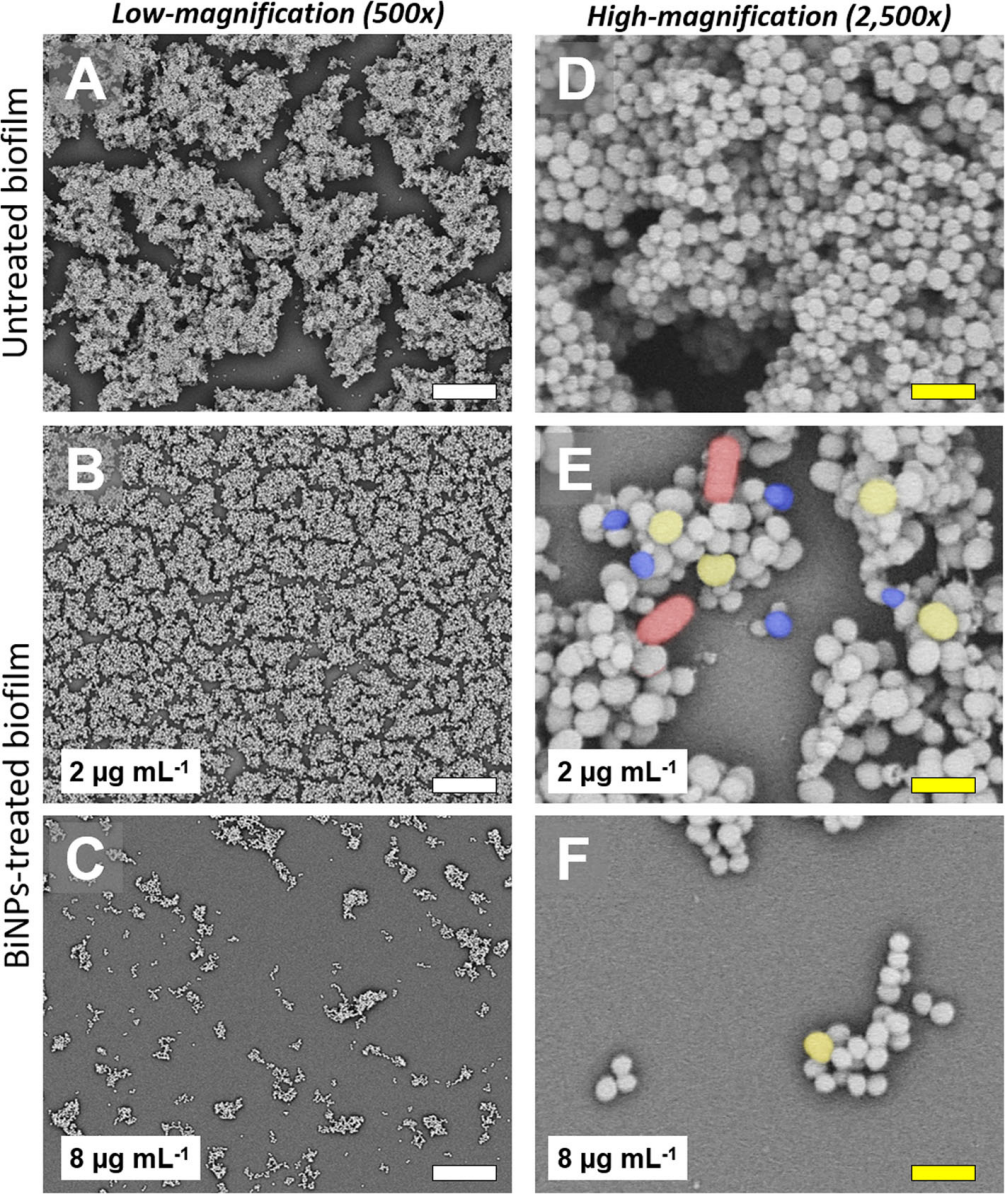
BiNPs inhibit biofilm formation by *S. aureus*. Untreated controls (**a**, **d**), as well as BiNPs-treated samples exposed to a low concentration of nanoparticles (**b**), form biofilms, but the cells from on the BiNPs-treated samples, display changes in morphology (**e**), from changes in size (yellow and blue-colored cells), and in shape (red-colored cells). Samples treated with a higher concentration of BiNPs do not form biofilms, but the cell morphology changes were less common (**f**). Scale bars: white = 20 μm, yellow = 2 μm

Scanning Electron Microscopy confirmed that BiNPs hinder the *S. aureus* ability to form biofilms. Low magnification SEM Images revealed that untreated *S. aureus* samples form biofilms with dense groups of cells (Fig. 5a). High-magnification SEM images confirmed the presence of highly-packed multi-layered clusters of cells (Fig. 5d). The morphology of the *S. aureus* cells revealed that they do not display any evident variations on their shape and size. In contrast, in the treated samples with sub-lethal concentrations of BiNPs (2 μg mL^− 1^), the treated biofilms appeared to be less dense, but with a larger covered area (Fig. 5b). The high-magnification images showed that the biofilm display lower thickness, with less clustered bacterial cells. Interestingly, the bacterial cell morphology was altered by the BiNPs, as evidenced by the diversity of cellular sizes and shapes observed (Fig. 5e). This may be caused by the effects of bismuth on the cells, such as the denaturing of proteins [68, 69] and changes in the cell membrane permeability [70]. Other potential mechanisms remain to be further studied and elucidated. In samples treated with higher concentrations of BiNPs (8 μg mL^− 1^), the biofilms formation was almost completely abolished. Some sparse, small clusters of bacterial cells were present (Fig. 5c), with most of the observed cells displaying similar shape and size, with the exception of a few larger cells (Fig. 5f).

**Fig. 6:**
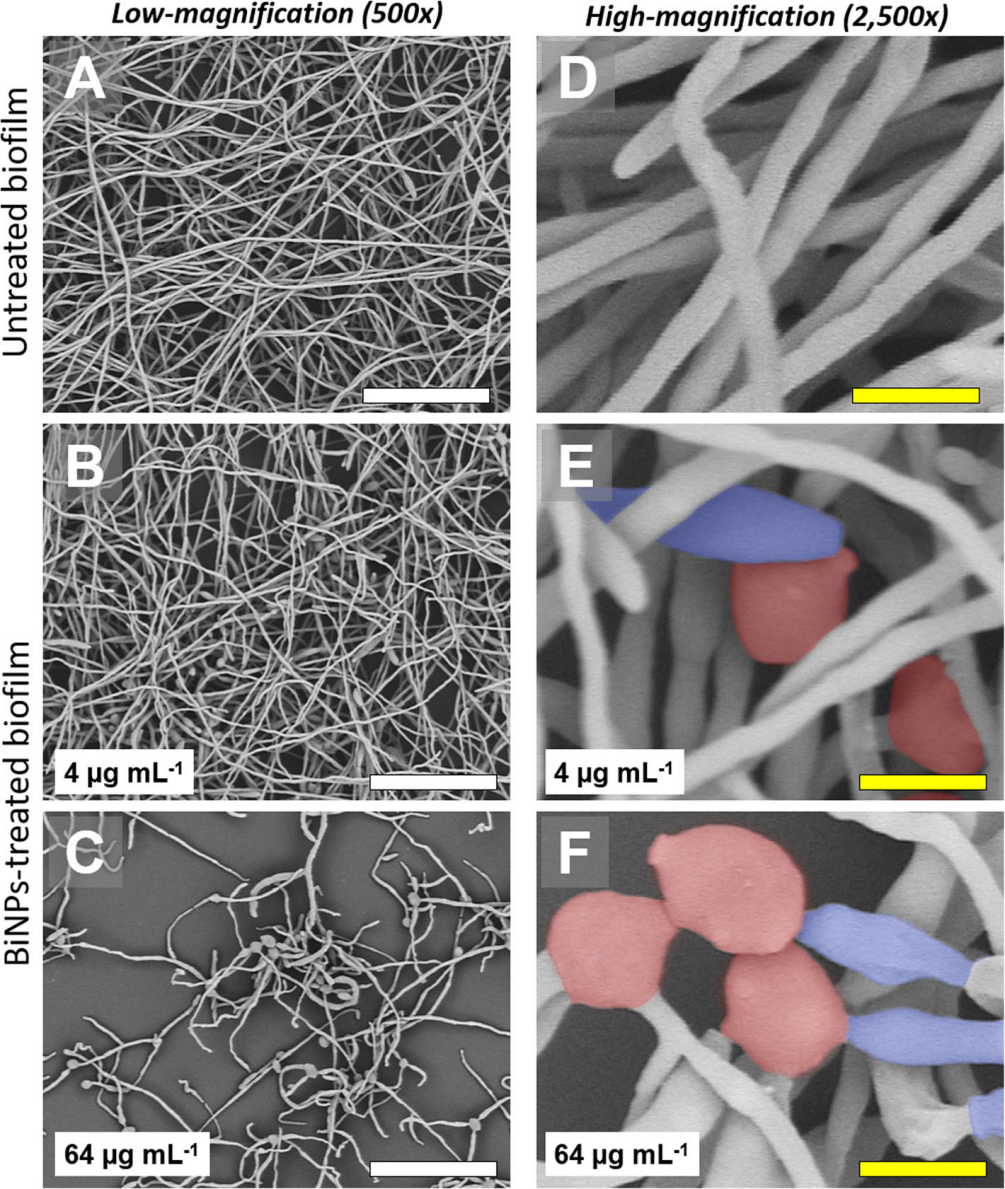
BiNPs affect the dimorphic transition and inhibit biofilm formation in *C. albicans*. Untreated controls (**a**, **d**) show the typical filamentous *C. albicans* biofilms; however, in samples treated with a low concentration of BiNPs, the dimorphic transition is affected. **b**, **e** In samples treated with a high concentration of BiNPs, biofilm formation is mostly inhibited, and the cells show aberrant morphologies (**c**, **f**). Yeast-like (in red) and pseudohyphae-like (in blue) cells were observed. Scale bars: white = 40 μm, yellow = 4 μm

Regarding the effect on *C. albicans* biofilms, SEM analysis confirmed that BiNPs negatively impact the dimorphic transition, from yeast to hyphae, and its ability to establish biofilms. On the untreated samples, low-magnification images revealed thick biofilms with fully-formed hyphae that completely cover the surface (Fig. 6a). High-magnification images showed that only the hyphal shape is visible in the samples (Fig. 6d). Samples treated with sub-lethal concentrations of BiNPs (4 μg mL^− 1^), also showed thick biofilms that totally covered the surface (Fig. 6b); however, alterations on the cell structure were evident. High-magnification images showed that yeast-like and pseudohyphae-like structures were abundant, confirming that BiNPs hinder the dimorphic transition ability of *C. albicans* (Fig. 6e). The impact of bismuth nanoparticles on the ability of *C. albicans* to form biofilms became more evident in samples treated with higher concentrations of BiNPs (64 μg mL^− 1^). At this concentration, biofilm formation was greatly inhibited by the BiNPs, as seen in Fig. 6c, where only a few, aberrant-shaped hyphae were observable. The yeast-like and pseudohyphae-like structures also presented alterations on their morphology (Fig. 6f).

## Conclusion

Our small, spheroid PVP-coated bismuth nanoparticles are more stable than the bismuth-BAL complex in aqueous solutions. Also, these BiNPs display antimicrobial activity against the bacterium *S. aureus* and the yeast *C. albicans* under both planktonic and biofilm formation stages. The antimicrobial activity of these BiNPs is comparable to or better than other BiNPs synthesized by more sophisticated methods. Moreover, the antimicrobial activity of the BiNPs is parallel to the commercially available antibiotics and is also similar in potency to that of silver nanoparticles. Microscopy analysis reveals that BiNPs reduce biofilm formation and negatively alter the cell morphology in both *S. aureus* and *C. albicans*. However, literature shows that bismuth nanoparticles may display cytotoxicity, therefore, more research regarding BiNPs biocompatibility and safety is needed for future applications. This work shows that cost-effective, fast, easy-to-synthesize nanomaterials may display broad antimicrobial activity against bacteria and fungi. These nanomaterials could be applied as broad-range sanitizers to decrease the dispersion of potential pathogenic cells and for reducing their ability to form biofilms in healthcare-related facilities and other public spaces.

## Supplementary information

**Additional file 1: Fig. S1.** TEM images confirms the presence of BiNPs. PVP-BiNPs display an aspect ratio close to 1 and the average size is below 10 nm. Occasional other shapes and sizes are also observed. **Fig. S2.** Bismuth nanoparticles in aqueous solutions remain stable over time. Visual examination reveals that BiNPs remained stable over time. Visually, an 11-week-old synthesis (B) kept light-protected at 4 oC, appears identical to a freshly prepared synthesis (A). UV-Vis spectrophotometry reveals that their optical properties (absorbance profiles) remain similar -with minor changes- (C) between the old synthesis (red) and the new preparation (blue). **Fig. S3.** Inhibition of S. aureus and C. albicans by different bismuth compounds under both planktonic and biofilm growing conditions. The dose-response curves against S. aureus (top panels), and C. albicans (bottom panels) show that bismuth compounds display different degrees of antimicrobial activity under planktonic (A, C) and biofilm (B, D) growing conditions. Lines: green (BiNPs), red (bismuth-BAL) and blue (Bi(NO3)3).

## Data Availability

Raw data from the “antimicrobial susceptibility assays” is available from the authors upon request.

## Abbreviations

PVP: Polyvinylpyrrolidone
BiNPs: Bismuth nanoparticles
MIC: Minimum Inhibitory Concentration
SEM: Scanning Electron Microscopy
BAL: British Anti-Lewisite; dimercaptopropanol.
