# Supplementary material for: Bismuth nanoparticles obtained by a facile synthesis method exhibit antimicrobial activity against Staphylococcus aureus and Candida albicans
Source: BMC Biomed Eng. 2020 Oct 14;2:11. doi: 10.1186/s42490-020-00044-2 (PMC7558697; doi:10.1186/s42490-020-00044-2)
Supplement: Supplementary file 1 — Additional file 1: Fig. S1. TEM images confirms the presence of BiNPs. PVP-BiNPs display an aspect ratio close to 1 and the average size is below 10 nm. Occasional other shapes and sizes are also observed. Fig. S2. Bismuth nanoparticles in aqueous solutions remain stable over time. Visual examination reveals that BiNPs remained stable over time. Visually, an 11-week-old synthesis (B) kept light-protected at 4 oC, appears identical to a freshly prepared synthesis (A). UV-Vis spectrophotometry reveals that their optical properties (absorbance profiles) remain similar -with minor changes- (C) between the old synthesis (red) and the new preparation (blue). Fig. S3. Inhibition of S. aureus and C. albicans by different bismuth compounds under both planktonic and biofilm growing conditions. The dose-response curves against S. aureus (top panels), and C. albicans (bottom panels) show that bismuth compounds display different degrees of antimicrobial activity under planktonic (A, C) and biofilm (B, D) growing conditions. Lines: green (BiNPs), red (bismuth-BAL) and blue (Bi(NO3)3). [file 42490_2020_44_MOESM1_ESM.docx]

**Bismuth nanoparticles obtained by a facile synthesis method
exhibit antimicrobial activity against *Staphylococcus aureus* and *Candida albicans***

**SUPPLEMENTARY MATERIALS**

**Figure S1**


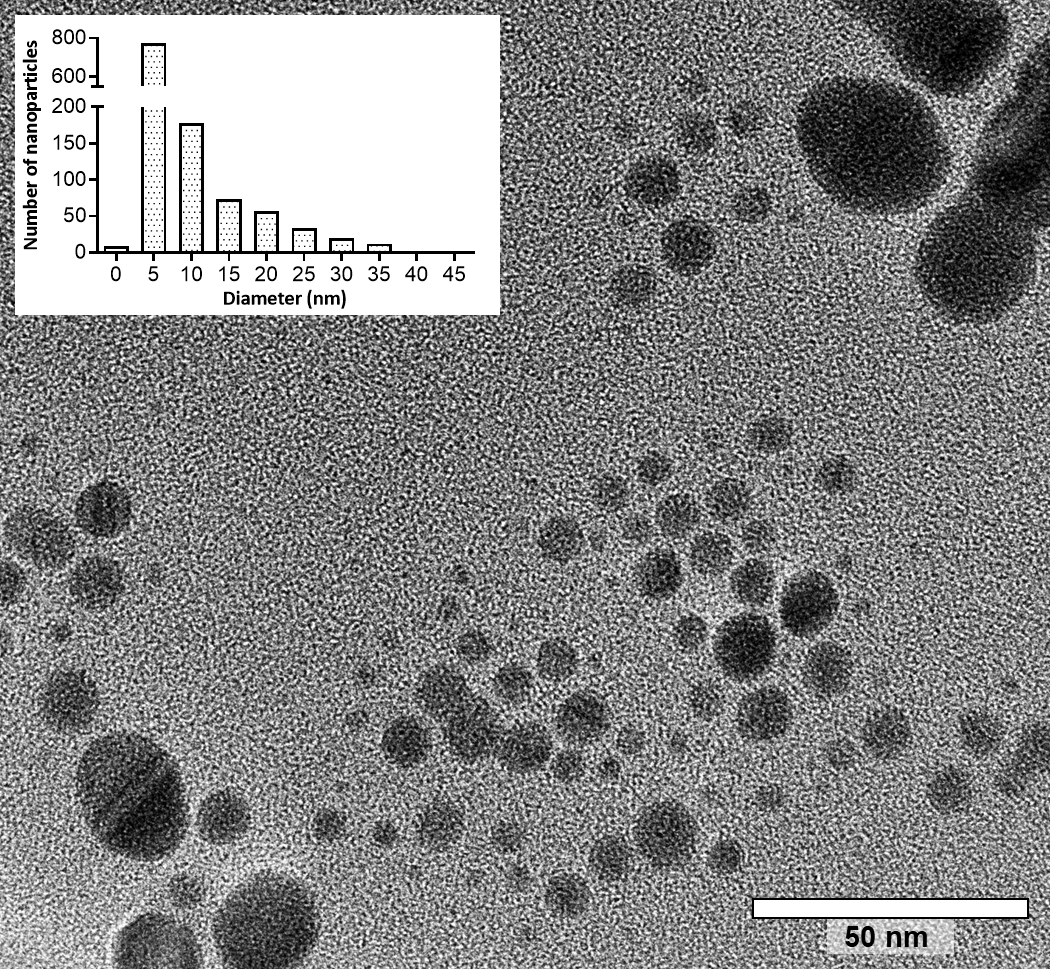


**Fig. S1.** **TEM images confirms the presence of BiNPs**. PVP-BiNPs display an aspect ratio close to 1 and the average size is below 10 nm. Occasional other shapes and sizes are also observed.

**Figure S2.**


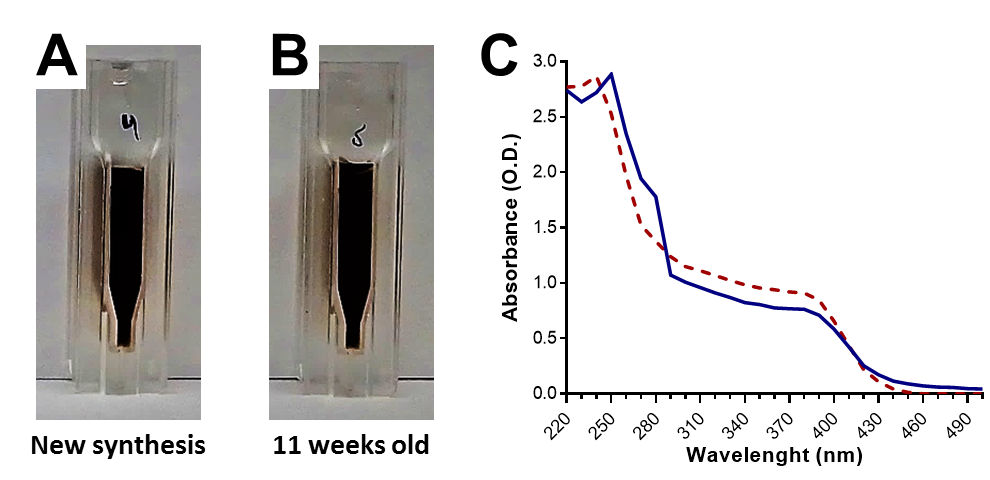


**Fig. S2. Bismuth nanoparticles in aqueous solutions remain stable over time**. Visual examination reveals that BiNPs remained stable over time. Visually, an 11-week-old synthesis (B) kept light-protected at 4 ^o^C, appears identical to a freshly prepared synthesis (A). UV-Vis spectrophotometry reveals that their optical properties (absorbance profiles) remain similar -with minor changes- (C) between the old synthesis (red) and the new preparation (blue).

**Figure S3**


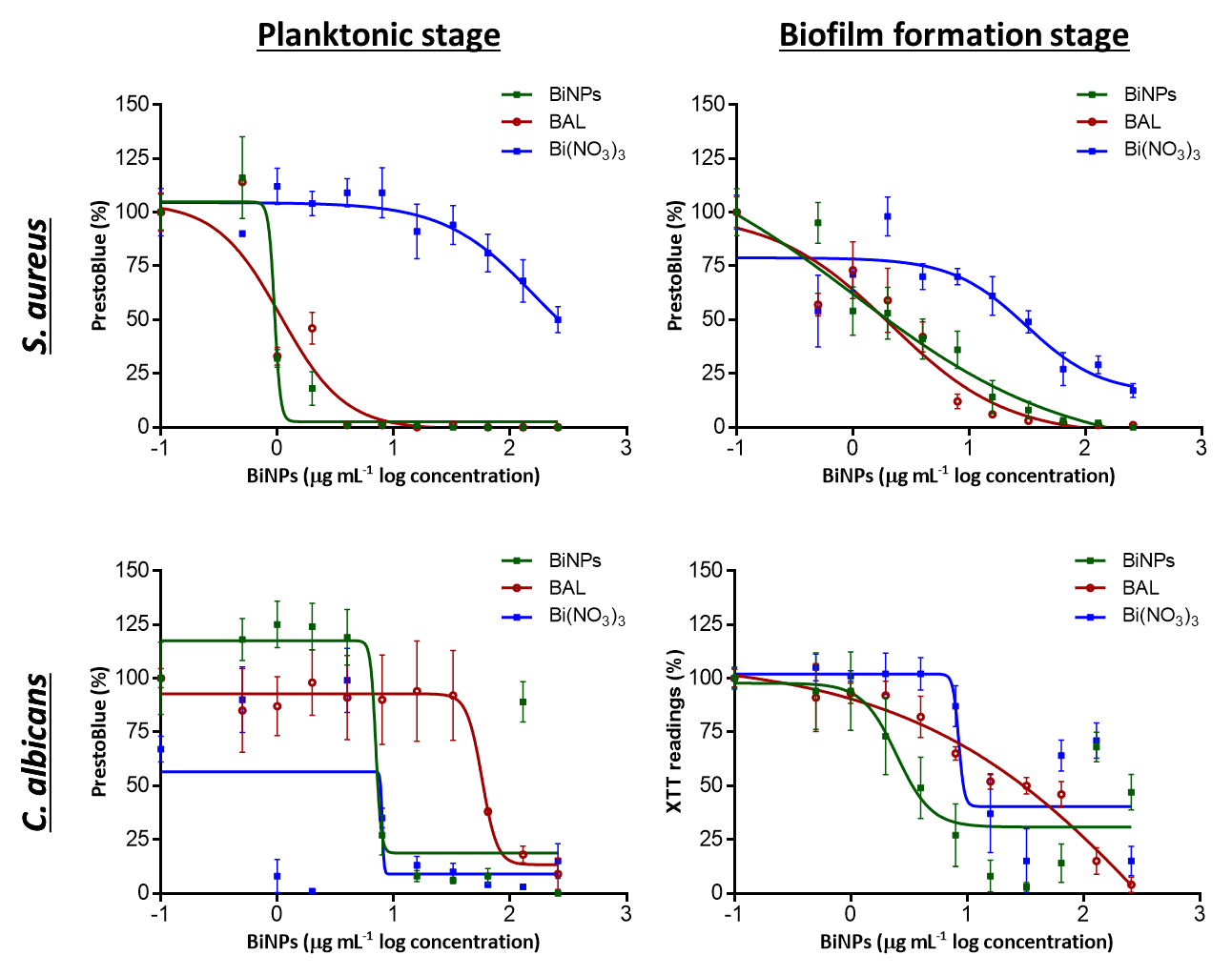


**Fig. S3. Inhibition of *S. aureus* and *C. albicans* by different bismuth compounds under both planktonic and biofilm growing conditions.** The dose-response curves against *S. aureus* (top panels), and *C. albicans* (bottom panels) show that bismuth compounds display different degrees of antimicrobial activity under planktonic (left) and biofilm (right) growing conditions. Lines: green (BiNPs), red (bismuth-BAL) and blue (Bi(NO_3_)_3_).

**Table S1.**

BiNPs Inhibitory Concentration (IC) against planktonic and biofilm stages of bacteria and fungi.

| **Stage /Strain** | | | **MIC**  **(µg mL^-1^)*** | **Reference** |
| --- | --- | --- | --- | --- |
| **Planktonic stage** | Bacteria | *S. mutans* | 1.05 | (1) |
|  |  | *S. aureus* UAMS-1 | 1 | **This work** |
|  |  | *S. aureus* ATCC 9182 | 128 / 256 | (2) |
|  |  | MRSA | 1,500 | (3) |
|  | Fungi | *C. albicans* ATCC 90029 | 2.08 | (1) |
|  |  | *C. albicans* ATCC SC5314 | 16 | **This work** |
|  |  | *C. albicans* ATCC 90029 | 416 | (4) |
| **Biofilm stage** | Bacteria | *S. aureus* UAMS-1 | IC_50_=1.06 | **This work** |
|  |  | MRSA | IC_84_=1,500 | (3) |
|  | Fungi | *C. albicans* ATCC SC5314 | IC_50_=7.9 | **This work** |

**References**

1. Badireddy AR, Hernandez-Delgadillo R, Sánchez-Nájera RI, Chellam S, Cabral-Romero C. Synthesis and characterization of lipophilic bismuth dimercaptopropanol nanoparticles and their effects on oral microorganisms growth and biofilm formation. J Nanoparticle Res [Internet]. 2014 Jun 29;16(6):2456. Available from: http://link.springer.com/10.1007/s11051-014-2456-5

2. Ma L, Wu J, Wang S, Yang H, Liang D, Lu Z. Synergistic antibacterial effect of Bi2S3nanospheres combined with ineffective antibiotic gentamicin against methicillin-resistant Staphylococcus aureus. J Inorg Biochem [Internet]. 2017;168:38–45. Available from: http://dx.doi.org/10.1016/j.jinorgbio.2016.12.005

3. Firouzi Dalvand L, Hosseini F, Moradi Dehaghi S, Siasi Torbati E. Inhibitory Effect of Bismuth Oxide Nanoparticles Produced by Bacillus licheniformis on Methicillin-Resistant Staphylococcus aureus Strains (MRSA). Iran J Biotechnol [Internet]. 2018 Nov 1 [cited 2019 May 8];16(4):279–86. Available from: http://www.ijbiotech.com/article_80169.html

4. Hernandez-Delgadillo R, Velasco-Arias D, Martinez-Sanmiguel JJ, Diaz D, Zumeta-Dube I, Arevalo-Niño K, et al. Bismuth oxide aqueous colloidal nanoparticles inhibit Candida albicans growth and biofilm formation. Int J Nanomedicine. 2013;8:1645–52.
